# OSVE or multiple-choice test: Is that a relevant question?

**DOI:** 10.1016/j.clinsp.2024.100519

**Published:** 2025-01-27

**Authors:** Francine Jomara Lopes, Renato Fraga Righetti, Matheus Belloni Torsani, Gustavo Silva Azevedo, Fernando Mauad Sacramento, Iolanda de Fátima Lopes Calvo Tibério

**Affiliations:** a Hospital Sírio-Libanês, São Paulo, São Paulo, SP, Brazil; b Department of Clinical Medicine, Faculdade de Medicina, Universidade de São Paulo (FMUSP), São Paulo, SP, Brazil

**Keywords:** OSCE, Virtual OSCE, Medical students, Medical Education, Virtual Medicine, COVID-19

## Abstract

**Introduction:**

The OSCE has been a robust tool for assessing clinical skills in medical education, adaptable across various clinical domains and stages. The COVID-19 pandemic, however, posed significant challenges to traditional assessment methods. This led to the development of the OSVE as an alternative.

**Objective:**

This study aimed to correlate OSVE results with MCT scores and Grade Point Average (GPA) of clerkship.

**Methods:**

This cross-sectional study involved 129 clerkships. It compared two OSVEs and two MCTs, covering content from the 5th and 6th years of the curriculum, against final graduation grades.

**Results:**

The score of GPA was 8.13 ± 0.43. The authors compared OSVE-5th (7.13 ± 0.93) and OSVE-6th (7.23 ± 0.83, NS), MCT-5th (7.08 ± 0.91) vs. MCT-6th (7.68 ± 1.21, *p* < 0.001), OSVE-5th (7.13 ± 0.93) and MCT-5th (7.08 ± 0.91, NS), OSVE-6th (7.23 ± 0.83) and MCT-6th (7.68 ± 1.21, *p* < 0.001) OSVE-5th (7.13 ± 0.93) and MCT-6th (7.68 ± 1.21, *p* < 0.001), OSVE-6th (7.23 ± 0.83) and MCT-5th (7.08 ± 0.91, NS), OSVE-5th (7.13 ± 0.93) and GPA (8.13 ± 0.43, *p* < 0.001), OSVE-6th (7.23 ± 0.83) and GPA (8.13 ± 0.43, *p* < 0.001), MCT-5th (7.08 ± 0.91) and GPA (8.13 ± 0.43, *p* < 0.001), and MCT-6th (7.68 ± 1.21) and GPA (8.13 ± 0.43, *p* < 0.001). There were positive correlations between OSVE-5th and OSVE-6th (*R* = 0.418, *p* < 0.05), OSVE-5th and MCT-5th (*R* = 0.475, *p* < 0.05), MCT-5th, OSVE-6th and MCT-6th (*R* = 0,180, *p* < 0.05), MCT-6th (*R* = 0.227, *p* < 0.05), with OSVE-6th and MCT-5th (*R* = 0.478, *p* < 0.05), OSVE-5th and GPA (*R* = 0.481, *p* < 0.05), OSVE-6th and GPA (*R* = 0,439, *p* < 0.05), MCT-5th and GPA (*R* = 0.681, *p* < 0.05) and MCT-6th and GPA (*R* = 0.195, *p* < 0.05), no correlation was found between OSVE-6th and MCT-6th (*R* = 0.070, NS).

**Conclusions:**

The correlations between the assessments revealed mild to moderate, suggesting that the assessments can be used interchangeably in the context of formative assessment. These results indicate the feasibility of using both OSVE and multiple-choice tests as effective tools to enhance intern training and monitoring**.**

## Highlights

• OSVE and multiple-choice tests can be used interchangeably in the context of formative assessment.
• OSVE and multiple-choice tests as effective tools to enhance intern training and monitoring.
• OSVE holds promise as a transformative tool, capable of ensuring the continuity and integrity of clinical skills assessment in medical education.

## Introduction

The process of evaluation in education is complex, involving diverse beliefs, values, principles, and theories. Particularly in medical education, evaluation aligns theoretical frameworks with practical applications and yields insights that can affirm or challenge the credibility of what is being assessed.^1^

Despite the logical progression in evaluation methodologies, the alignment of strategies, procedures, instruments, and objectives with desired criteria and contextual factors is not always straightforward. Underneath the surface of assessment lies a web of meanings that requires careful consideration.^1^

Educational evaluation serves dual purposes: formative assessment, which facilitates learning; and summative assessment, which gauges achievement and enables progression. Assessments typically fall into three domains: knowledge (cognitive aspects), skills (psychomotor aspects), and attitudes (affective aspects), shaping competency-based learning.^2^, ^3^, ^4^, ^5^

The reliability, validity, and applicability of assessment instruments are crucial, regardless of the chosen method for evaluating clinical skills, knowledge, attitudes, and postures.^6^ The OSCE has been a popular and flexible tool in this regard, adapting to various educational stages and resource availability.^7^, ^8^, ^9^, ^10^, ^11^ However, the COVID-19 pandemic's restrictions necessitated a shift in assessment approaches, giving rise to the OSVE as an alternative.^12^^,^^13^ This study investigates the effectiveness of the OSVE in comparison to traditional MCTs and Grade Point Average (GPA) in the context of medical education's rapidly evolving landscape.

## Methods

### Subjects and setting

This study was structured according to Strengthening the Reporting of Observational Studies in Epidemiology (STROBE). This analytical cross-sectional study compared the Objective structured Video Examination (OSVE) with Multiple-Choice Test (MCT) and Grade Point Average (GPA). Conducted at the University of São Paulo School of Medicine, which admits 175 students annually to its six-year undergraduate medical program, this research focused on the clerkship experience during the final two years. Students are exposed to a range of clinical environments, including a 258-bed secondary hospital and a 1200-bed tertiary hospital, covering pediatrics, internal medicine, obstetrics and gynecology, surgery, and preventive medicine.

### Data collection

The study included 129 students who successfully completed their assessments to reach the sixth year in the internal medicine clerkship in 2021. Data collection was approved by the Ethics Committee (Ethics Committee study protocol number 5.102.186) and involved extracting information from the medical school database. The assessments included two OSVEs and two MCTs, focusing on the 5th and 6th year curriculum content, alongside the final grade.

### Analysis

Statistical analysis utilized SPSS Version 20, Minitab 16, and Excel Office 2010. The significance level was set at 5 %, with confidence intervals at the 95 % level. Non-parametric statistical tests assessed the normality of the primary outcome variables. The Wilcoxon test for paired data, tests for equality of two proportions, and Pearson's correlation coefficient were employed in the analysis.

The design of the OSVE and MCT was based on a blueprint established by a team of professors and preceptors from the internship program, which was reviewed by three specialists in education. For the OSVE, scenarios and checklists were developed, followed by simulations among team members for adjustments in language, content, and time for completion. The technical aspects of the application program, such as sound and image quality, were also checked. The MCT followed a similar process, excluding the filming and technical processes inherent to the OSVE. The GPA was calculated as the average of all curricular units and internships, representing the overall academic performance of the students throughout the course, and the final scores for the OSVE and MCT were used interchangeably in the selection process.

## Results

**Fig. 1:**
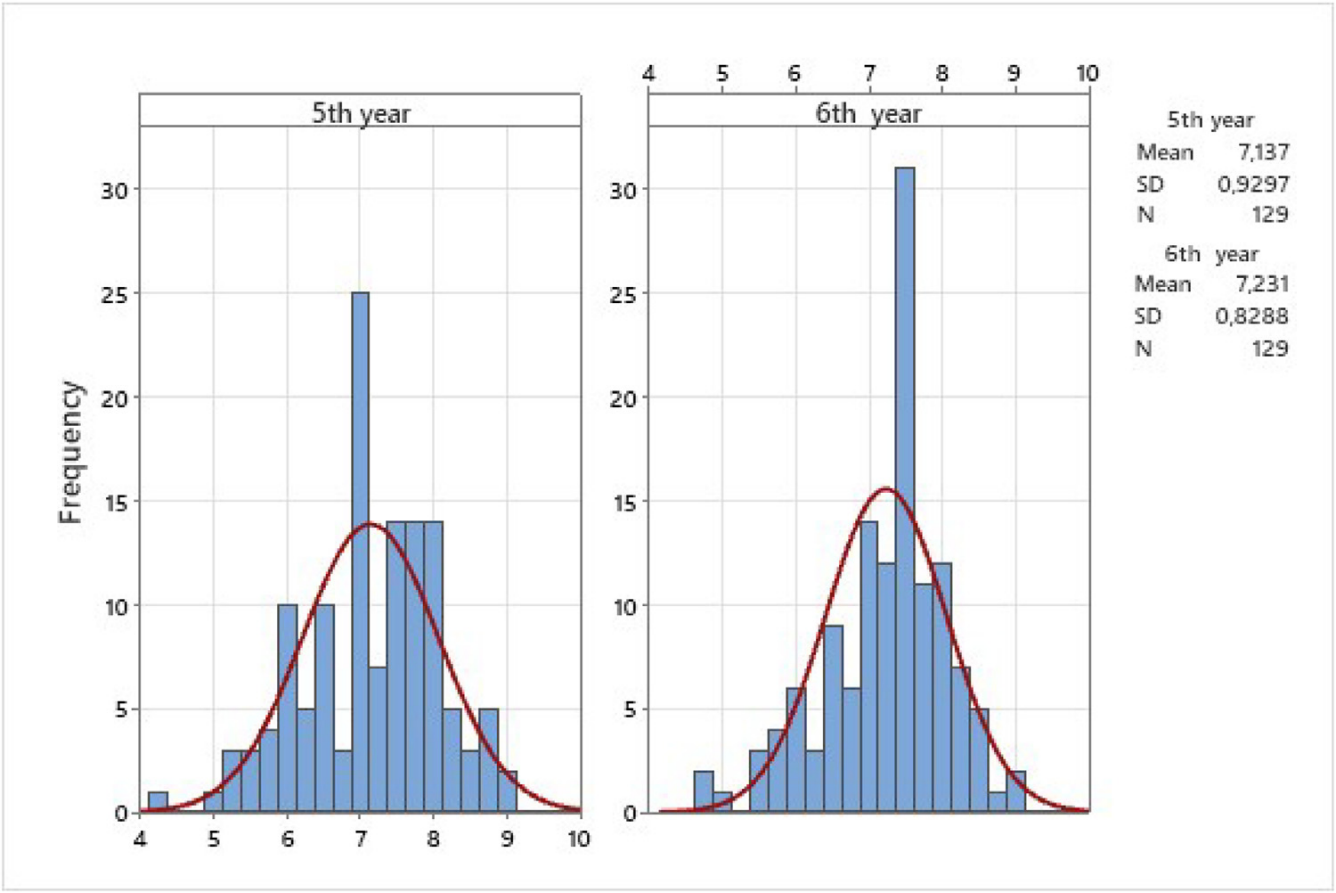
Histogram of distribution of averages in OSVE in both applications. 2023.

**Fig. 2:**
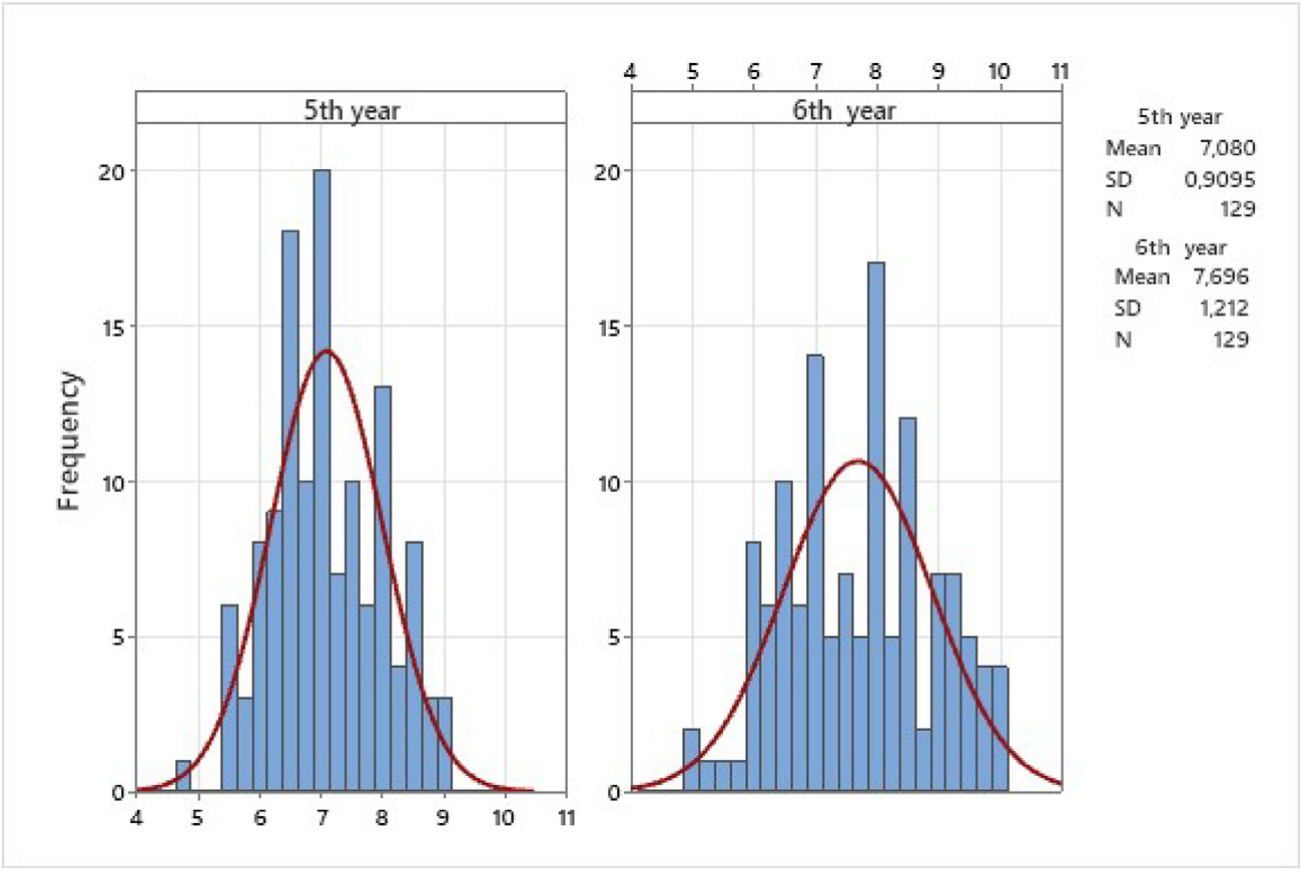
Histogram of distribution of averages in multiple-choice test in both applications. 2023.

The study observed comparable averages between the two OSVE applications and the multiple-choice tests. However, a slight increase was noted in MCT scores. The grade point average (FG) was 8.13 ± 0.43. Notable comparisons included OSVE-5th (7.13 ± 0.93) and MCT-5th (7.08 ± 0.91, NS), indicating no significant differences. Correlation analyses revealed varying degrees of positive correlations between OSVE and MCT scores, suggesting a mild to moderate relationship in assessment outcomes. Table 1 shows the analysis of variance and comparisons of assessment methods and Table 2 shows the descriptive statistics of five medical main areas. Fig. 1, Fig. 2 show histograms of the distribution of averages in OSVE and MCT for both applications.

**Table 1:** Analysis of variance and comparisons of assessment methods. 2023.

	Mean ± SD	OSVE-5Th	OSVE-6Th	MCT-5Th	MCT-6Th
OSVE-5Th	7137 ± 0.930	‒	‒	‒	‒
OSVE-6Th	7231 ± 0.829	NS	‒	‒	‒
MCT-5Th	7080 ± 0.910	NS	NS	‒	‒
MCT-6Th	7696 ± 1212	*p* < 0.001	*p* < 0.001	*p* < 0.001	‒
GPA	8137 ± 0.438	*p* < 0.001	*p* < 0.001	*p* < 0.001	*p* < 0.001

OSVE, Objective Structured Video Examination; MCT, Multiple-Choice Test; GPA, Grade Point Average.

**Table 2:** Descriptive statistics of five main areas: Surgery (SUR) Internal Medicine (IM), Preventive Medicine (MP) Obstetrics and Gynecology (OG), and Pediatrics (PED). 2023.

	OSVE-5Th	OSVE-6Th	MCT-5Th	MCT-6Th
SUR	8.174 ± 1.628	5.907 ± 1.817	7.736 ± 1.173	8.415 ± 1.233
IM	7.302 ± 1.540	7.841 ± 0.948	6.965 ± 1.372	7.461 ± 1.638
MP	8.477 ± 1.066	6.027 ± 1.803	7.240 ± 1.027	7.473 ± 1.629
OG	6.267 ± 1.513	8.174 ± 1.419	6.140 ± 1.595	7.659 ± 1.528
PED	5.585 ± 1.972	8.353 ± 1.209	7.426 ± 1.141	7.508 ± 1.413

SUR, Surgery; IM, Internal Medicine; MP, Preventive Medicine; OG, Obstetrics and Gynecology; PED, Pediatrics.

Fig. 1, Fig. 2 show histograms of the distribution of averages in OSVE and MCT for both applications.

## Discussion

The Objective Structured Clinical Examination (OSCE) has long been the gold standard in assessing clinical skills within medical education, allowing for a robust evaluation across various stages of learning. However, the advent of the COVID-19 pandemic has necessitated the exploration of alternative, remote assessment methods. This has led to the emergence of the Objective Structured Video Examination (OSVE), a novel modality that has the potential to revolutionize the assessment of clinical skills in the absence of traditional, in-person evaluations.^2^

Recent studies have expressed growing concerns regarding the future of medical education post-pandemic, with a focus on the suitability of assessments for physical examination skills.^14^^,^^15^ There is a widespread belief that the execution of physical examinations may be compromised in virtual environments. An illustrative example is a study by Shaban et al.^16^ that investigated the difference between medical students before and during the pandemic. The results of this study revealed that the pandemic had a significant and detrimental impact on the learning performance of final-year medical students, particularly in their clinical experience.

The present study compared the efficacy of OSVE with that of traditional Multiple-Choice Tests (MCTs) for assessing medical students during their clerkships, also comparing with GPA (Grade Point Average). The study included 129 medical students in a public medical school, evaluating their performance on the subjects of two academic years using both assessment methods. The present findings revealed that while OSVE scores closely mirrored the final grades of clerkships, suggesting a comprehensive assessment of students' clinical abilities, the MCTs did not consistently reflect these competencies.

Shaban et al.^17^ discovered that OSVE is a highly effective alternative when compared to traditional OSCE. This is evident in the substantial reduction in students' stress and anxiety levels. Prettyman et al.^18^ observed several benefits related to student learning, including effective assessment of clinical competencies by instructors, all achieved with minimal resource utilization.

The Grade Point Average (GPA) was 8.13±0.43, which was significantly higher. This is partly due to the fact that, in addition to assessments, GPA also includes the evaluation of performance during the clerkship, which tends to be higher than the averages of exam scores.

The correlation analyses between OSVE and MCT scores provided insightful revelations. OSVE scores for subjects from the 5th and 6th years showed a positive correlation, indicating consistency in evaluating clinical competencies over time. Moreover, there was a significant positive correlation between OSVE scores and GPA, emphasizing the potential of OSVE to serve as a reliable predictor of students' overall performance in their clinical education.

However, the study also highlighted some challenges associated with the implementation of OSVE, such as the need for significant resources and the skepticism of educators regarding its effectiveness over MCTs. Despite these challenges, the potential educational benefits of OSVE cannot be overlooked. The flexibility of OSVE in assessing various clinical domains, its feasibility during times when traditional assessments are impractical, and its ability to provide a more dynamic and interactive assessment experience position it as a valuable tool in the medical educators' repertoire.

The resources required for the implementation of OSVE are often available within the educational institution itself, such as technology (computers with cameras and microphones and internet access). Students use their own devices and internet for the assessments, which appears to provide a cost advantage. This is in contrast to traditional OSCE, which can be more expensive due to the need for human and physical resources.^18^^,19^

In the analysis of data derived from two OSVE applications, which recorded scores of 7.13±0.93 and 7.23±0.83, along with two multiple-choice test administrations yielding scores of 7.08±0.91 and 7.68±1.21, a comparative evaluation was conducted. This assessment aimed to examine the consistency of student performance during the clerkship. The results indicated comparable average performance across both instances of the OSVE applications and the multiple-choice tests, with a noticeable, albeit slight, increase in the average scores of the multiple-choice tests. Additionally, the Pearson correlation analysis of these grades demonstrated low to moderate correlation coefficients, ranging from 0.195 to 0.681, with a statistical significance of *p*< 0.05. This finding suggests a variable degree of association between the different evaluative methods and the student's academic performance as indicated by these assessments.

Given that the correlations of the evaluation of students in undergraduate courses are low to moderate, it is recommended that both OSVE and MCTs be used complementarily during the assessment of students throughout their course. Additionally, both assessment methods can be effectively utilized in selection processes, such as residency exams. Considering that there is no significant difference between the average scores of both methods, either can be appropriately employed for this purpose.

A brief comparative analysis was conducted of the five main areas (surgery, internal medicine, preventive medicine, obstetrics and gynecology, and pediatrics). The clerkships achieved higher average scores in both the OSVE and the multiple-choice tests when students were exposed to the 6th year of the curriculum. However, in the case of OSVE, a contrasting pattern emerged in the fields of surgery and preventive medicine, where the highest averages were actually recorded during the 5th year of the curriculum. This slight fluctuation in the averages may be attributed to variations in the complexity of the questions.

This study contributes to the ongoing dialogue about the effectiveness of various assessment methods in medical education. It underscores the need for further research and development in virtual assessment tools like OSVE, particularly in times when conventional methods are constrained. As medical education continues to evolve, so must assessment strategies. Objective structured video examination holds promise as a transformative tool, capable of ensuring the continuity and integrity of clinical skills assessment in medical education, even in the face of unprecedented global challenges.

### Study limitations

The present study was conducted at a single center with specific educational settings. Therefore, it is important to emphasize that the results obtained cannot be widely generalized and should be validated through additional studies conducted in different countries and educational centers. This broader approach would allow us to gain a more comprehensive understanding of the utility of OSVE as a safe and effective method for formative assessment. The authors recommend that future research explore the comparison between assessment methods in various educational contexts to enrich understanding of the topic.

It is important to acknowledge the limitations of the OSVE in comparison to the traditional OSCE. The OSVE primarily assesses students' ability to recall and memorize information, such as clinical signs, rather than their ability to seek information and demonstrate practical techniques in unpredictable situations. This limitation is also shared with the MCT, suggesting that both assessments might be equivalent in this regard.

## Conclusion

The analysis of the correlation between the assessments revealed mild to moderate correlations, suggesting that the assessments can be used interchangeably in the context of formative assessment. These results indicate the feasibility of using both OSVE and multiple-choice tests as effective tools to enhance intern training and monitoring.

## Declaration of competing interest

The authors declare no conflicts of interest.

## References

[1] Marinho-Araujo CM Rabelo M.L. (2015). Avaliação educacional: a abordagem por competências. Avaliação (Campinas).

[2] Haydar A., Siqueira M.A.M., Torsani M.B., Tibério I.F.L.C. Avaliação de habilidades clínicas. In: Martins MA, Quintino CR, Tibério IFLC, Atta JA, Ivanovic LF. Semiologia Clínica. São Paulo: Manole; 2021. p. 553–64.

[3] Boucher F.G., Palmer W.H., Page G., Barriault R., Seely J. (1980). The evaluation of clinical competence. Can Fam Phys.

[4] Prislin M.D., Fitzpatrick C.F., Lie D., Giglio M., Radecki S., Lewis E. (1998). Use of an objective structured clinical examination in evaluating student performance. Fam Med.

[5] Kemahli S. (2001). Clinical teaching and OSCE in pediatrics. Med Educ Online.

[6] Harden R.M (1979). How to assess clinical competence-an overview. Med Teach.

[7] Rodrigues M.A.V., Olmos R.D., Kira C.M., Lotufo P.A., Santos I.S. (2019). Tibério IFLC. “Shadow” OSCE examiner. A cross-sectional study comparing the “shadow” examiner with the original OSCE examiner format. Clinics.

[8] Rodrigues M.A.V.M. (2019).

[9] Al-Balas M., HI Al-Balas, Jaber H.M., Obeidat K., Al-Balas H., Aborajooh E.A. (2020). Correction to: distance learning in clinical medical education amid COVID-19 pandemic in Jordan: current situation, challenges, and perspectives. BMC Med Educ.

[10] Troncon L.E.A., Foss N.T., Voltarelli J.C., Dantas R.O. (1996). Avaliação de habilidades clínicas por exame objetivo estruturado por estações, com emprego de pacientes padronizados: descrição de dois métodos (Parte I). Rev Bras Educ Med.

[11] Zarifsanaiey N., Amini M., Saadat F. (2016). A comparison of educational strategies for the acquisition of nursing student's performance and critical thinking: simulation-based training vs. integrated training (simulation and critical thinking strategies). BMC Med Educ.

[12] Silverman J.A., Foulds J.L. (2020). Development and use of a virtual objective structured clinical examination. Can Med Educ J.

[13] Updike W.H., Cowart K., Woodyard J.L., Serag-Bolos E., Taylor J.R., Curtis S.D. (2021). Protecting the integrity of virtual objective structured clinical examination. Am J Pharm Educ.

[14] Chan S.C.C., Choa G., Kelly J., Maru D., Rashid M.A. (2023). Implementation of virtual OSCE in health professions education: a systematic review. Med Educ.

[15] Tzeng T.Y., Hsu C.A., Yang Y.Y., Yuan E.J., Chang Y.T., Li T.H. (2022). The Impact of COVID-19 Pandemic on the learning outcomes of medical students in Taiwan: a two-year prospective cohort study of OSCE performance. Int J Environ Res Public Health.

[16] Shaban S., Tariq I., Elzubeir M., Alsuwaidi A.R., Basheer A., Magzoub M. (2021). Conducting online OSCEs aided by a novel time management web-based system. BMC Med Educ.

[17] Shaiba L.A., Alnamnakani M., Temsah M.H., Alamro N., Alsohime F., Alrabiaah A. (2021). Medical faculty's and students' perceptions toward pediatric electronic OSCE during the COVID-19 pandemic in Saudi Arabia. Healthcare.

[18] Prettyman A.V., Knight E., Allison T.E. (2018). Objective structured clinical examination from virtually anywhere!. J Nurse Practition.

